# Mutant dominant-negative *rhodopsin* ^*∆I256*^ causes protein aggregates degraded via ERAD and prevents normal rhodopsin from proper membrane trafficking

**DOI:** 10.3389/fmolb.2024.1369000

**Published:** 2024-05-17

**Authors:** Bowen Cao, Johanna Valentina Dahlen, Merve Sen, Tina Beyer, Tobias Leonhard, Ellen Kilger, Blanca Arango-Gonzalez, Marius Ueffing

**Affiliations:** ^1^ Centre for Ophthalmology, Institute for Ophthalmic Research, University of Tübingen, Tuebingen, Germany; ^2^ Graduate Training Centre of Neuroscience, University of Tübingen, Tuebingen, Germany

**Keywords:** rhodopsin, ADRP, dominant-negative effect, VCP, ERAD, proteasome, retinitis pigmentosa

## Abstract

Dominant mutations in the rhodopsin gene (*Rho*) contribute to 25% of autosomal dominant retinitis pigmentosa (adRP), characterized by photoreceptor loss and progressive blindness. One such mutation, *Rho* ^*∆I256*^ *,* carries a 3-bp deletion, resulting in the loss of one of two isoleucines at codons 255 and 256. Our investigation, using recombinant expression in HEK293 and COS-7 cells, revealed that *Rho* ^*∆*I256^, akin to the known adRP mutation *Rho* ^P23H^, induces the formation of rhodopsin protein (RHO) aggregates at the perinuclear region. Co-expression of *Rho* ^*∆I256*^ or *Rho* ^*P23H*^ with wild-type *Rho* ^*WT*^, mimicking the heterozygous genotype of adRP patients, demonstrated the dominant-negative effect, as all isoforms were retained in perinuclear aggregates, impeding membrane trafficking. In retinal explants from WT mice, mislocalization of labeled adRP isoforms at the outer nuclear layer was observed. Further analysis revealed that RHO^∆I256^ aggregates are retained at the endoplasmic reticulum (ER), undergo ER-associated degradation (ERAD), and colocalize with the AAA-ATPase escort chaperone valosin-containing protein (VCP). These aggregates are polyubiquitinated and partially colocalized with the 20S proteasome subunit beta-5 (PSMB5). Pharmacological inhibition of proteasome- or VCP activity increased RHO^∆I256^ aggregate size. In summary, RHO^∆I256^ exhibits dominant pathogenicity by sequestering normal RHO^WT^ in ER aggregates, preventing its membrane trafficking and following the ERAD degradation.

## 1 Introduction

Retinitis pigmentosa (RP) is a progressive rod-cone dystrophy caused by the dysfunction and eventual death of rod photoreceptors, followed by cone photoreceptor cell death. Initial symptoms of this disease include impaired dark adaptation and “night blindness” (nyctalopia), followed by a loss of peripheral vision and, in advanced cases, possible tunnel vision. As the disease progresses, central vision is also affected (Hartong et al., 2006). RP is currently incurable and is the leading cause of visual impairment and blindness in individuals under the age of 60 (Cross et al., 2022). While clinical therapy cannot reverse the damage caused by RP, efforts are focused on slowing down visual deterioration, treating complications, and helping patients cope with the psychosocial impact of their condition (Frick et al., 2012).

The rhodopsin gene (*Rho*) was the first gene identified to cause RP. This gene encodes Rhodopsin protein (RHO), a seven-transmembrane G-protein-coupled receptor expressed exclusively in mammalian rod photoreceptors. RHO is synthesized and folded in the endoplasmic reticulum (ER) and processed in the Golgi (Deretic, 2006). Proper folding and trafficking of RHO is essential for normal phototransduction and rod cell survival. The fully functional form of RHO consists of the apoprotein opsin, which binds the chromophore 11-cis retinal (Hargrave, 2001; Pearring et al., 2013). RHO accounts for approximately 90% of all proteins in the discs. Its absence prevents outer segment (OS) formation and leads to photoreceptor cell death (Berson, 1996; Humphries et al., 1997; Lem et al., 1999; Rakshit and Park, 2015).

*Rho*-mediated RP can be inherited in an autosomal dominant (adRP) or autosomal recessive (arRP) pattern. In the adRP cases, over 150 mutations in the *Rho* gene have been identified, accounting for 20%–30% of all adRP cases (Athanasiou et al., 2018). Researchers have attempted to categorize these mutations based on their biochemical and cellular properties (Inglehearn et al., 1991; Kaushal and Khorana, 1994; Mendes et al., 2005; Krebs et al., 2010; Rakoczy et al., 2011). Interestingly, most Rho mutations fall into class II, characterized by protein misfolding, ER retention, and OS instability (Athanasiou et al., 2018).

A 3-base pair (bp) deletion in the human *Rho* gene, deleting one of the two isoleucine (I) at codon 255 or 256 in the sixth transmembrane domain (referred to in this paper as *Rho* ^*∆I256*^), has been suggested to be the major cause of adRP in Europe (Inglehearn et al., 1991; Artlich et al., 1992). *Rho* ^*∆I256*^ was first discovered in a British family (Inglehearn et al., 1991) and later found in German, Belgian, and Chinese families (Lorenz et al., 1993a; Li et al., 2010). Patients with this mutation show clinical symptoms and signs of RP with an early onset of nyctalopia, a constricted visual field, and even reduced visual acuity or color vision abnormalities (Lorenz et al., 1993a; Li et al., 2010). However, the biochemical changes or associated molecular mechanisms that link the *Rho* ^*∆I256*^ mutation to photoreceptor cell death are still not fully understood. According to Sung *et al.*, *Rho* ^*∆I256*^ causes biochemical defects associated with class II mutations, with RHO^∆I256^ protein localizing and accumulating in the ER (Sung et al., 1993). However, the mechanisms underlying how this mutation leads to photoreceptor cell death remain unclear.

It has been reported that cells with dominant *Rho* mutations undergo cell death as a result of toxic gain-of-function (GOF) and/or dominant-negative (DN) activity (Athanasiou et al., 2018). GOF mechanisms include protein misfolding, aggregation formation, and unfolded protein response (UPR) induction. On the other hand, DN effects of dominant *Rho* mutations may eliminate one or more functions associated with normal RHO, affecting the maturation, trafficking, and activity of wild-type RHO (RHO^WT^). RP caused by the DN mechanisms may be due to RHO aggregates that interfere with endogenous RHO maturation (Wilson and Wensel, 2003). These two mechanisms are supported by studies of the *Rho* ^*P23H*^ (proline to histidine substitution at position 23) class II mutation (Illing et al., 2002; Saliba PMM et al., 2002; Rajan and Kopito, 2005; Griciuc, 2010). *Rho* ^*P23H*^ is the most common *Rho* mutation in North America and arguably the most studied one, accounting for 10% of all cases of adRP in the US in patients of Western European origin (Sullivan et al., 2006). The *Rho* ^*P23H*^ mutant forms ER-bound aggregates and interferes with the UPR system. In addition, these aggregates prevent the proper processing of RHO^WT^.

According to several studies, *Rho* ^*∆I256*^ and *Rho* ^*P23H*^ exhibit similar phenotypes (Dryja et al., 1990; Lorenz et al., 1993b; Naash et al., 1993; Sung et al., 1993; Kaushal and Khorana, 1994; Daiger et al., 2007), probably because mutations affecting *Rho* transmembrane or intradiscal domain residues are generally classified as class II mutations (Lorenz et al., 1993b), suggesting that *Rho* ^*∆I256*^ may share a similar pathogenetic mechanism with *Rho* ^*P23H*^. In previous studies, we could show that RHO^P23H^ aggregate formation is coupled to ER-associated protein degradation (ERAD) (Griciuc et al., 2010a), a major protein processing pathway that is divided into four steps: substrate recognition, ubiquitination, retrotranslocation to the cytosol, and proteasome-mediated degradation (Guerriero and Brodsky, 2012). Further, we found that misfolded RHO^P23H^ is targeted by valosin-containing protein (VCP), a type II AAA ATPase chaperone that processes and earmarks RHO^P23H^ to be transferred from the endoplasmic reticulum (ER) to the proteasome system for degradation (Wang et al., 2004). VCP is the driving force for the retrotranslocation of ER-retained aggregates from the ER to the cytosol for proteasomal degradation (Ye et al., 2001). Studies have shown that VCP interacts with aggregates or ubiquitin-positive inclusions in patients with neurodegenerative diseases (Mizuno et al., 2003; Boeddrich et al., 2006; Gitcho et al., 2009). Our previous research has also shown that inhibition of VCP activity suppresses retinal pathology and preserves retinal structure and function in *Rho* ^*P23H*^-associated adRP animal models (Griciuc et al., 2010b; Arango-Gonzalez et al., 2020; Sen et al., 2021a; Sen et al., 2021b). Here, we show that, similar to *Rho* ^*P23H*^, degradation of RHO^∆I256^ aggregates undergoes a VCP-dependent ERAD process. Most importantly, we find that RHO^∆I256^-containing cytoplasmic aggregates retain RHO^WT^ and interfere with the normal localization or trafficking of RHO^WT^ to the cell membrane, which explains its dominant-negative mechanism. (All abbreviations are shown in Supplementary Table S1).

## 2 Results

### 2.1 *Rho* ^*∆I256*^ mutant is mislocalized and tends to form high molecular weight aggregates in the cytosol

**FIGURE 1:**
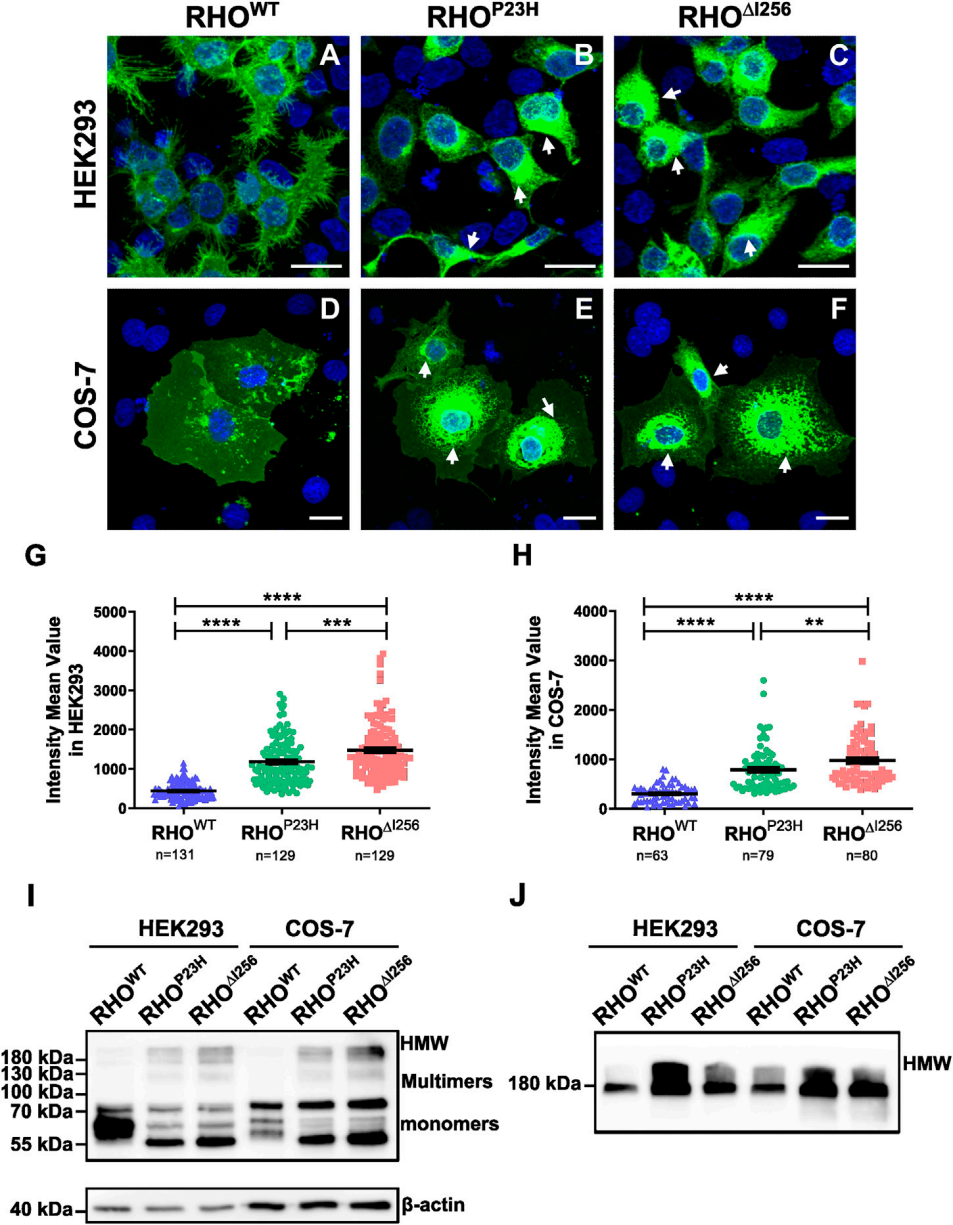
RHO^∆I256^ mislocalizes to the cytosol and forms high molecular weight aggregates *in vitro*. Fluorescence images of HEK293 **(A–C)** and COS-7 cells **(D–F)** transfected with *Rho* ^*WT*^ *-*EGFP, *Rho* ^*P23H*^-EGFP, or *Rho* ^*∆I256*^-EGFP (green). RHO^WT^-EGFP is predominantly localized at the plasma membrane, while RHO^∆I256^-EGFP (white arrows in C, F) and RHO^P23H^-EGFP (white arrows in B, E) accumulate in the cytosol. Scale bar: 20 μm. There was a significant increase in the mean fluorescence intensity (MFI) in cells transfected with *Rho* ^*∆I256*^-EGFP or *Rho* ^*P23H*^-EGFP cells compared to *Rho* ^*WT*^-EGFP **(G–H)**. Immunoblot analysis revealed that RHO^∆I256^-EGFP forms HMW aggregates in both soluble **(I)** and insoluble cell fractions **(J)**. Data were expressed as mean ± SEM. Significance was calculated by Mann-Whitney-U-test. **p < 0.01, ***p < 0.001, ****p < 0.0001.

To study the cellular behavior of *Rho* ^*∆I256*^ *in vitro*, we transfected different *Rho* constructs (*Rho* ^*WT*^, *Rho* ^*P23H*^, or *Rho* ^*∆I256*^), tagged with EGFP using the pEGFP-N1 vector, into HEK293 and COS-7 cells (Figure 1). As expected, RHO^WT^-EGFP was correctly targeted and evenly distributed to the plasma membrane in both cell types, with only scattered RHO^WT^-EGFP puncta in the cytosol, which is consistent with previous results (Figure 1A, D) (Illing et al., 2002; Saliba PMM et al., 2002; Griciuc et al., 2010a). In contrast, most RHO^∆I256^ was confined to the perinuclear region, with high fluorescence intensity (Figure 1C, F), similar to the cellular localization of RHO^P23H^ (Figure 1B, E). We measured the mean fluorescence intensity (MFI) in the cytoplasmic region for all three groups to quantify RHO aggregation. We found that the MFI of RHO was significantly increased in both *Rho* ^*∆I256*^-EGFP and *Rho* ^*P23H*^-EGFP transfected cells (HEK293 cells: 1,477 ± 695.9; COS-7 cells: 974.2 ± 503.3; HEK293 cells: 1,182 ± 570.9; COS-7 cells: 788.1 ± 445.8, respectively) compared to *Rho* ^*WT*^-EGFP transfected cells (HEK293 cells: 438.8 ± 207.9; COS-7 cells: 303.2 ± 181.6) (Figures 1G, H), indicating that *Rho* ^*∆I256*^, similar to *Rho* ^*P23H*^, leads to a high degree of RHO accumulation. We also found that *Rho* ^*∆I256*^-EGFP results in a significantly higher MFI than *Rho* ^*P23H*^-EGFP (HEK293 cells: *Rho* ^*∆I256*^ vs. *Rho* ^*P23H*^ *p* < 0.0001; COS-7 cells: *Rho* ^*∆I256*^ vs. *Rho* ^*P23H*^ *p* < 0.05, Mann-Whitney-U-test, at least 40 cells in HEK293 and 20 cells in COS-7 cells from three different experiment were quantified), suggesting that the *Rho* ^*∆I256*^ mutation induces more severe protein aggregation.

Given the potential influence of large EGFP tags on the structure, oligomerization, and aggregate formation of the target protein, we transfected HEK293 cells with pEGFP-N1 plasmids encoding only EGFP. We compared the behavior of this control group with that of cells transfected with *Rho* ^WT^-EGFP, *Rho* ^P23H^-EGFP, or *Rho* ^∆I256^-EGFP. Remarkably, cells transfected with the plasmid encoding only EGFP did not exhibit aggregate formation (Supplementary Figure S1), a finding consistent with previous studies (Griciuc et al., 2010a). To further investigate the component of the accumulated RHO aggregates in the cytoplasm, we monitored RHO levels in detergent-soluble (Figure 1I) and detergent-insoluble (Figure 1J) extracts from HEK293 cells that had been transiently transfected with *Rho* ^*WT*^-EGFP, *Rho* ^*∆I256*^-EGFP, or *Rho* ^*P23H*^-EGFP constructs using SDS-PAGE Western blotting. The mature monomeric form of RHO containing the EGFP tag was observed as a band at 55–75 kDa, whereas dimers and trimers appear as smears at approximately 130 kDa and 180 kDa, respectively. High molecular weight (HMW) species with molecular weights greater than 180 kDa indicated RHO-containing oligomers and aggregates. RHO^WT^-EGFP was mostly detected in soluble extracts (Figure 1I) and showed diffuse bands of the predominantly monomeric form of the mature RHO, with minor multimers or HMW RHO. These results suggest that most RHO^WT^-EGFP forms a native mature conformation (Griciuc et al., 2010a).

On the other hand, RHO^∆I256^-EGFP showed a completely different behavior. Most of the mutant RHO was found in dimers, trimers, and HMW oligomers in the soluble cell fraction (Figure 1I), indicating a non-native conformation or immature RHO since RHO is monomeric in its native form in the membrane (Downer and Cone, 1985). Furthermore, a significant amount of RHO^∆I256^-EGFP appeared as HMW species in the detergent-insoluble fractions (Figure 1J) compared to RHO^WT^-EGFP, suggesting that *Rho* ^*∆I256*^ is more likely to form RHO aggregates than RHO^WT^. Similar observations were also made for RHO^P23H^-EGFP.

In conclusion, our results suggest that *Rho* ^*∆I256*^, like the *Rho* ^*P23H*^ mutation, causes a mislocalization of RHO that cannot be effectively targeted to the plasma membrane. In addition, mutant RHO is more prone to form cytosolic non-native HMW aggregates than RHO^WT^.

### 2.2 Dominant *Rho* ^*∆I256*^ mutation impairs the localization of WT rhodopsin

Most, if not all, patients with adRP carrying the *Rho* ^*∆I256*^ mutation inherit one mutant allele and one normal (WT) allele (Inglehearn et al., 1991; Lorenz et al., 1993a; Li et al., 2010). To closely mimic the heterozygous nature of the human patient genotype, we co-transfected HEK293 and COS-7 cells with EGFP-tagged *Rho* ^*∆I256*^, *Rho* ^*P23H*^, or *Rho* ^*WT*^ and mcherry-tagged *Rho* ^*WT*^ at equal concentrations. We used the two fluorescent tags to visualize any colocalization or interaction between mutant RHO^∆I256^ and RHO^WT^.

**FIGURE 2:**
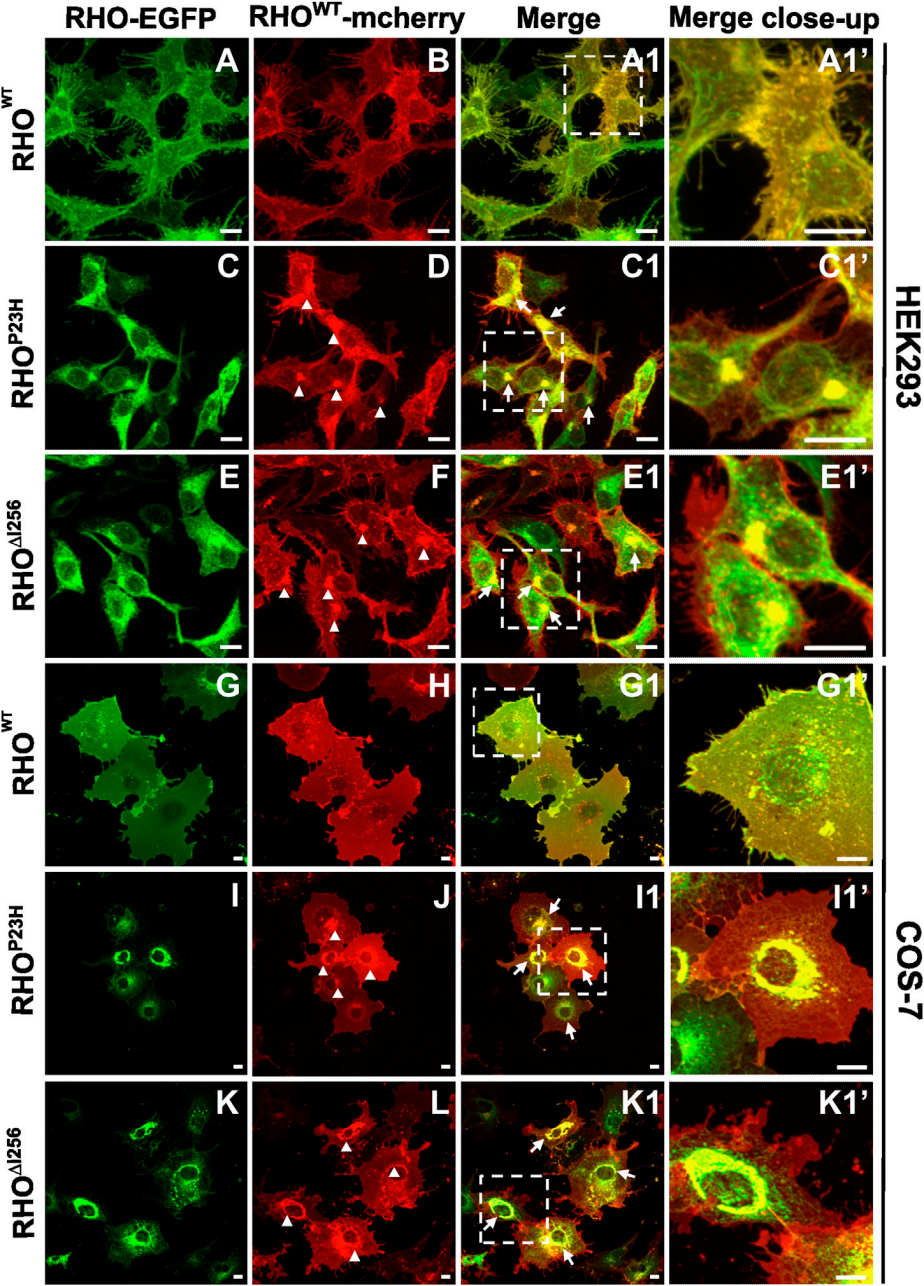
*Rho* ^*∆I256*^-EGFP and *Rho* ^*WT*^-mcherry co-expression causes intracellular retention of WT rhodopsin. Fluorescence microscopy detecting EGFP **(A, C, E, G, I, K)** and mcherry **(B, D, F, H, J, L)** in HEK293 and COS-7 cells co-transfected with *Rho* ^*WT*^-mcherry and *Rho* ^*∆I256*^-EGFP or *Rho* ^*P23H*^-EGFP plasmids. **(A1, C1, E1, G1, I1, and K1)** show the merged images of the EGFP and mcherry channels. Arrows indicate the colocalization of RHO^WT^-mcherry with RHO^∆I256^-EGFP or RHO^P23H^-EGFP. Magnified view is shown in **(A1', C1', E1', G1', I1', and K1')**. Arrowheads mark the mislocalized RHO^WT^ retained in misfolded aggregates **(D, F, J, L)**. Scale bar: 10 μm.

Co-transfection of *Rho* ^*∆I256*^ *-*EGFP and *Rho* ^*WT*^-mcherry in HEK293 and COS-7 cells resulted in intracellular mislocalization of RHO^∆I256^-EGFP as previously observed (Figure 2E, K). Here, RHO^WT^ *-*mcherry was also mislocalized to the perinuclear region and overlapped significantly with Rho^∆I256^-EGFP aggregates (Figure 2E1, K1). In comparison, when co-expressing both differently tagged *Rho* ^*WT*^ plasmids, RHO^WT^-EGFP and RHO^WT^-mcherry were predominantly localized to the plasma membrane (Figure 2A, B, G, H), as previously observed for RHO^WT^. These results indicate that RHO^∆I256^ impairs the correct membrane localization of the RHO^WT^ protein. Since a fraction of RHO^WT^-mcherry was still targeted to the cell membrane in cells co-transfected with mutant RHO, this suggests that the impairment of RHO^WT^ was not complete. Similar results were obtained in cells co-expressing RHO^WT^-mcherry with RHO^P23H^-EGFP (Figures 2I,J), indicating that the molecular pathomechanisms underlying the two dominant *Rho* mutations are likely similar.

Our results show that mislocalized RHO^∆I256^ aggregates trap part of RHO^WT^ in the aggregate pool, preventing RHO^WT^ from reaching its correct location in the cell membrane. As a result, a dominant-negative (DN) effect may play an important role in the pathology of *Rho* ^*∆I256*^-associated retinal degeneration (Herskowitz, 1987).

### 2.3 Proper targeting of endogenous WT rhodopsin to the photoreceptor outer segment is compromised by exogenously expressed RHO^∆I256^ in C57BL/6J retinal explants

The recruitment of RHO^WT^ into the RHO^∆I256^ aggregates in cells supports the hypothesis that the *Rho* ^*∆I256*^ allele causes RP via a DN mechanism. To investigate this, we transfected *Rho* ^*∆I256*^-EGFP into retinal explants from C57BL/6J (wild-type, WT) mice, which naturally produce RHO^WT^ that localizes to the rod outer segments (ROS). To determine whether RHO^∆I256^ affects endogenous RHO^WT^ localization, we used reverse magnetofection, a technique to deliver *Rho* ^*∆I256*^-EGFP into the C57BL/6J retina (Gomez-Tourino et al., 2013; Sen et al., 2021a; Bassetto et al., 2021). We labeled total RHO with a specific red fluorescing dye conjugated antibody to differentiate between EGFP-tagged exogenous RHO (displayed fluorescence in both red and green) and endogenous RHO (exhibited only red fluorescence). The comprehensive details on the *Rho* ^*∆I256*^-EGFP transfection group as well as other transfection groups, are listed in Supplementary Table S2.

**FIGURE 3:**
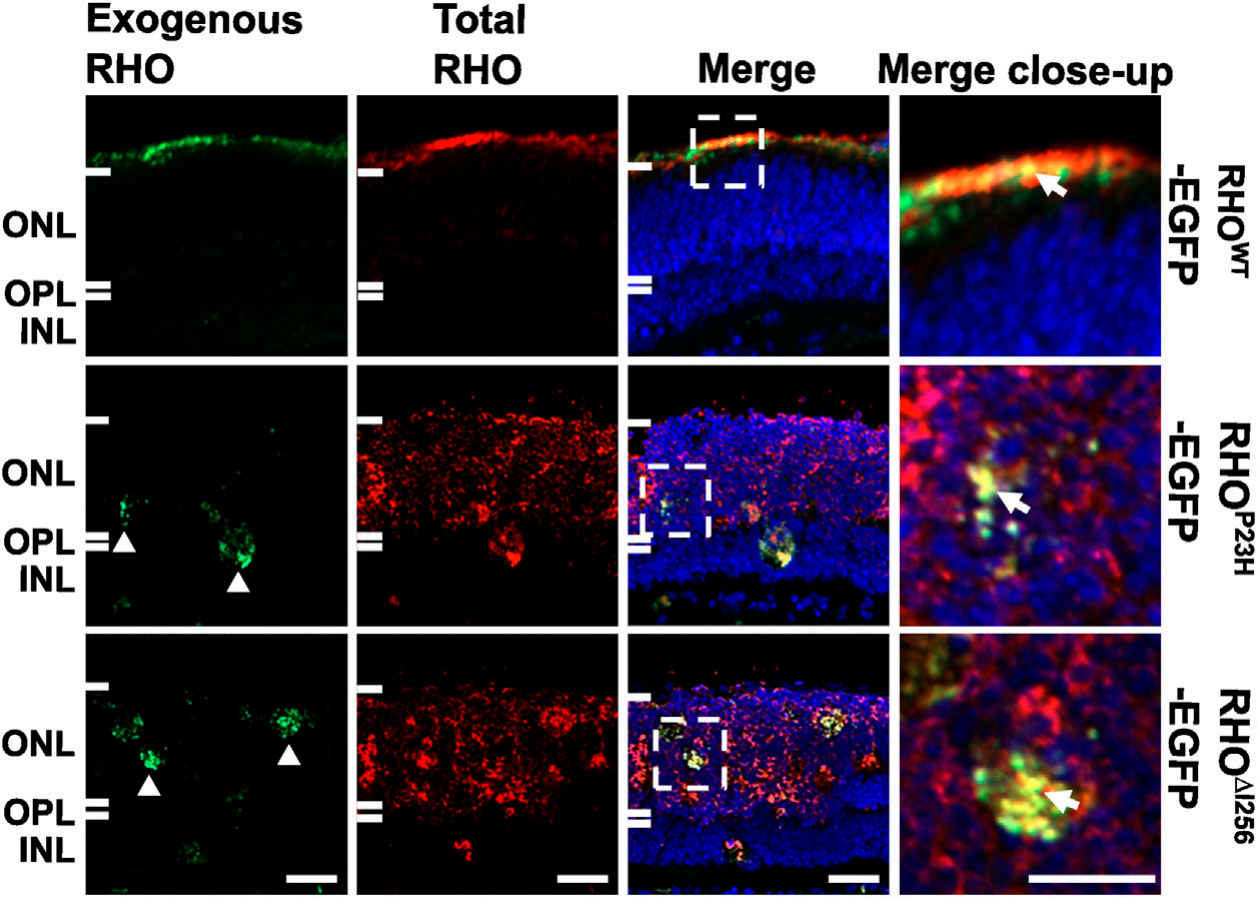
In C57BL/6J retinal explants, the trafficking of endogenous RHO^WT^ is affected by transfection with *Rho* ^*∆I256*^. Immunofluorescence pictures reveal the localization of total RHO (red, second column), including endogenously expressed RHO and EGFP-tagged exogenously expressed RHO in three experimental groups in C57BL/6J retinae transfected with *Rho* ^*WT*^-EGFP/XPMag (first row), *Rho* ^*P23H*^-EGFP/XPMag (second row), and *Rho* ^*∆I256*^-EGFP/XPMag (third row). Exogenously expressed RHO (green, first column) is indicated by EGFP fluorescence. Exogenous RHO^∆I256^-EGFP is mislocalized in the ONL (white arrowheads, green channel) and leads to the mislocalization of endogenous RHO^WT^ (red, third row). Moreover, in *Rho* ^*∆I256*^-EGFP/XPMag transfected C57BL/6J mouse retinal explants, mislocalized RHO^∆I256^-EGFP partially overlaps endogenous RHO^WT^ (white arrows, merged close-up), and the OS layer is thinner. In contrast, exogenous RHO^WT^-EGFP correctly targets the OS layer and colocalizes with endogenously expressed RHO^WT^ in transfected C57BL/6J mouse retinal explants. Scale bar: 20 μm.

When C57BL/6J retinal explants were transfected with *the Rho* ^*WT*^-EGFP/XPMag complex, both endogenous and exogenous RHO^WT^ correctly targeted the OS layer and largely overlapped each other. However, when the C57BL/6J retinal explants were transfected with the *Rho* ^*∆I256*^-EGFP/XPMag complex, a significant amount of endogenous RHO^WT^ was mislocalized and remained in the ONL instead of being properly targeted to the OS (Figure 3). As expected, exogenous RHO^∆I256^-EGFP was trapped in the ONL and overlapped with part of endogenous RHO^WT^. We observed the same result in the *Rho* ^*P23H*^-EGFP/XPMag-transfected C57BL/6J retinal explants. Furthermore, transfection of either of these two dominant *Rho* mutants into C57BL/6J retinal explants resulted in reduced OS length (Figure 3), which is a hallmark of retinal degeneration, indicating a degenerative effect (Milam et al., 1996; Sakai et al., 2003). Endogenous RHO was correctly targeted to the OS in all three control groups (complete medium CM, XPMag, or EGFP-transfected groups, as shown in Supplementary Figure S2).

Our findings support the hypothesis that the transport of endogenous RHO^WT^ is impaired in the presence of the dominant *Rho* ^*∆I256*^ (or *Rho* ^*P23H*^) mutant, suggesting that the DN effect plays an essential role in the pathophysiology of *Rho* ^*∆I256*^-associated adRP.

### 2.4 RHO^∆I256^ is retained at the ER and forms a complex with the ERAD effector VCP

Our results show that the dominant *Rho* ^*∆I256*^ mutation leads to cytoplasmic aggregates retaining WT rhodopsin from reaching its final physiological destination at the membrane. We and others have previously shown that such aggregates can be substrate to ERAD, the major degradation pathway for unfolded or misfolded protein aggregates. To determine whether RHO^∆I256^ aggregates are substrates of the ERAD clearance pathway, we analyzed whether they are tethered to ER sites and recognized by the ERAD effector VCP (Griciuc et al., 2010a; Sen et al., 2021b).

**FIGURE 4:**
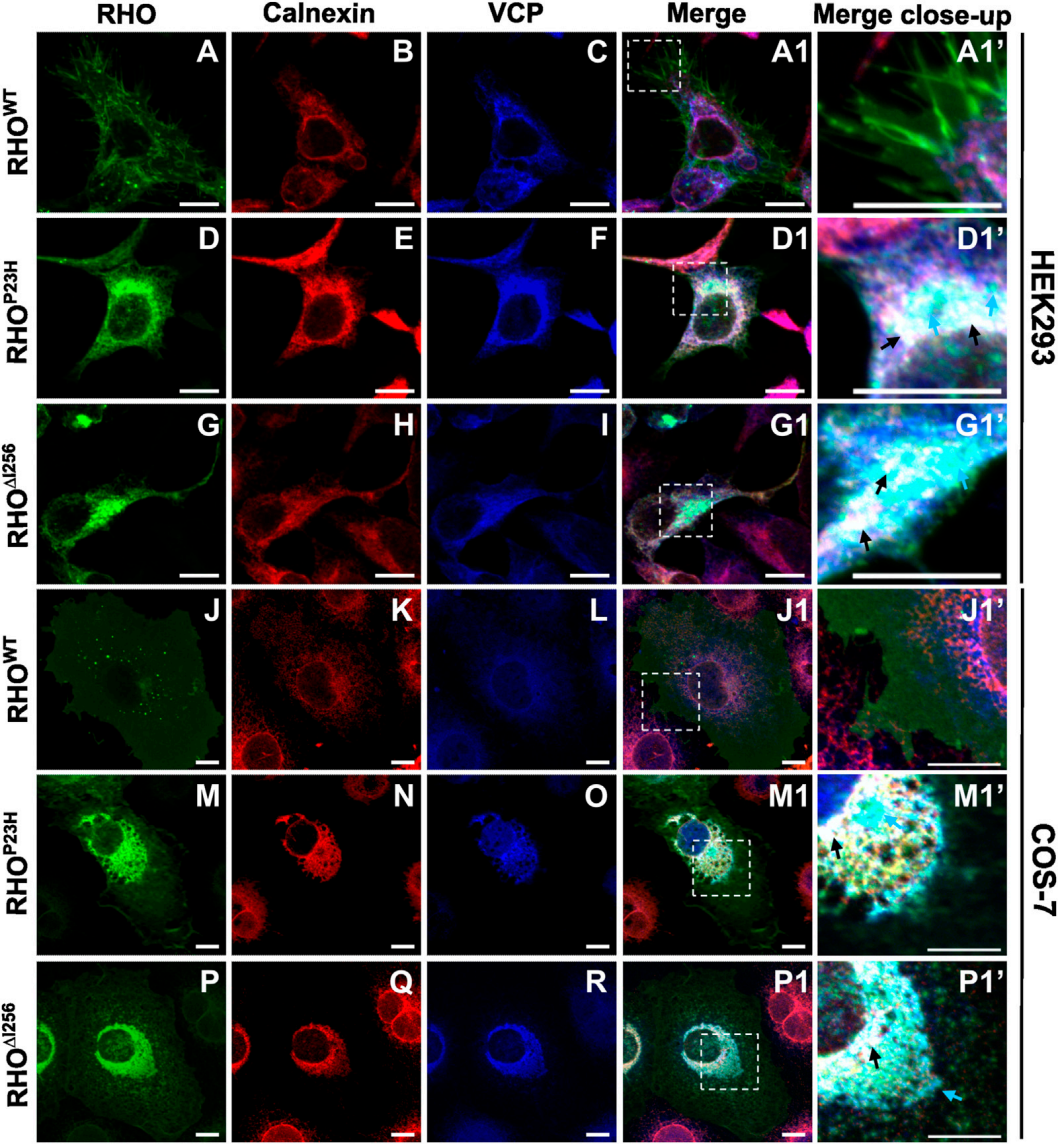
RHO^∆I256^ aggregates colocalize with ER and VCP. Immunofluorescence staining shows the localization of transfected *Rho*-EGFP (green, first column), the ER marker Calnexin (red, second column), and endogenous VCP (blue, third column) in HEK293 **(A–I, A1, D1, G1)** or COS-7 cells **(J–R, J1, M1, P1)**. Colocalization is revealed in merged pictures (fourth column), and higher magnification pictures from merged insets are shown in column 5. RHO^WT^ is mainly localized to the plasma membrane (A and J). RHO^∆I256^ aggregates localize with VCP to the ER (black arrows, **D1’, M1’**) and partially colocalize only with VCP (cyan arrows, **D1’, M1’**). RHO^P23H^ also accumulates in ER and VCP (black arrows, **G1’, P1’**). Scale bar: 10 μm.

We transfected HEK293 or COS-7 cells with plasmids encoding EGFP-tagged *Rho* ^*∆I256*^, *Rho* ^*P23H*^, or *Rho* ^*WT*^ and analyzed colocalization of RHO-EGFP with ER or VCP by immunofluorescence staining with calnexin- or VCP-specific antibodies. Our results revealed a partial colocalization between RHO^∆I256^ aggregates with ER and VCP in both cell lines (Figure 4). In *Rho* ^*WT*^-EGFP transfected cells, we hardly observed any colocalization of RHO^WT^ with calnexin or VCP (Figure 4A1’, J1’). In contrast, in *Rho* ^*∆I256*^-EGFP transfected cells, aggregated mutant RHO^∆I256^ partially colocalized with the ER marker calnexin and with VCP (Figure 4G1, P1 and black arrows in G1’, P1’). Furthermore, some of the RHO^∆I256^-containing cytoplasmic aggregates were not found within the ER but only co-stained with VCP, indicating that they were extracted from the ER and delivered to the cytosol by VCP. Similarly, the *Rho* ^*P23H*^ mutation led to the formation of aggregates that colocalized with calnexin and VCP, in agreement with our previous results (Griciuc et al., 2010a). Using Pearson’s correlation coefficient analysis, we further quantified the colocalization between RHO and ER or VCP (Supplementary Figure S3A, B). Compared with RHO^WT^ protein, RHO^∆I256^ and RHO^P23H^ aggregates showed significantly increased association with ER or VCP, which was consistent with the above immunostaining data.

The localization of endogenous VCP was observed predominantly in the cytoplasm in *Rho* ^*WT*^-EGFP-transfected cells (Figure 4C, L). However, in cells that express mutant RHO^∆I256^ (Figure 4I, R) or RHO^P23H^ (Figure 4F, O), the distribution of VCP mostly colocalized with calnexin, suggesting that the recruitment of VCP to the ER is involved in the retrotranslocation of aggregates.

### 2.5 Mislocalized RHO^∆I256^ aggregates are partially polyubiquitinated

It is known that almost all ERAD substrates are ubiquitinated prior to targeting the proteasome (Illing et al., 2002). In order to evaluate the role of ubiquitin (Ub) in the degradation of RHO^∆I256^ aggregates or RHO^WT^, we compared the changes in the localization and expression levels of ubiquitin proteins in *Rho* ^*∆I256*^-EGFP or *Rho* ^*WT*^-EGFP transfected cells using ubiquitin-specific antibodies. *Rho* ^*P23H*^-EGFP transfected cells were used as a positive control since RHO^P23H^ is much more ubiquitinated than RHO^WT^ (Illing et al., 2002).

**FIGURE 5:**
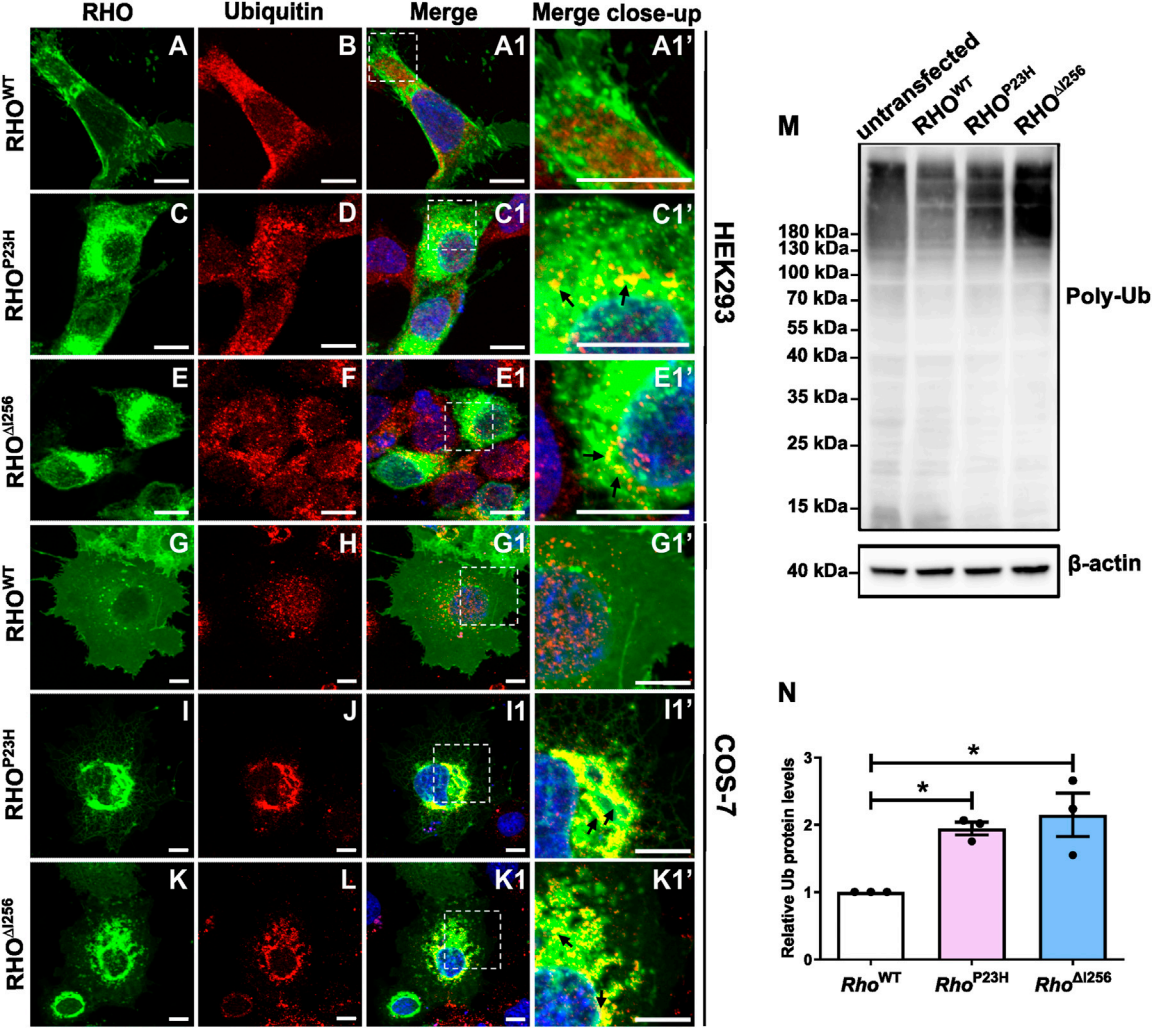
Misfolded RHO^∆I256^ is ubiquitinated. **(A–L)** Immunofluorescence of EGFP-tagged RHO^WT^, RHO^∆I256^, or RHO^P23H^ (green, first column) and Ub (red, second column) in HEK293 **(A–F, A1, A1’-E1, E1’)** and COS-7 cells **(G–L, G1, G1’-K1, K1’)**. Colocalization is shown in merged pictures (third column). Ub is recruited into the aggregates pool, which is revealed as yellow dots in merged pictures in *Rho* ^*∆I256*^ (black arrows, **E1’, K1’**) or *Rho* ^*P23H*^ transfected cells (black arrows, **C1’, I1’**). Scale bar: 10 μm; **(M)**. Western blot results show enhanced smear Ub bands in cell lysates of *Rho* ^*∆I256*^ or *Rho* ^*P23H*^ compared to *Rho* ^*WT*^-transfected HEK293 cells. Ub expression is also present in untransfected control group. β-actin is used as housekeeping marker. **(N)**. Quantification of Ub expression. Data are expressed as mean ± SEM. Statistical significance was determined by one-way ANOVA followed by Bonferroni’s multiple comparison test (*p < 0.05). Three independent experiments were included in the data analysis.

We found that RHO^∆I256^ aggregates were strongly ubiquitinated in transfected HEK293 and COS-7 cells colocalizing with endogenous ubiquitin (Figure 5). In both cell types transfected with *Rho* ^*WT*^-EGFP, ubiquitin was not specifically colocalized with rhodopsin, yet distributed throughout the whole cytosol (Figure 5B, H). No enhanced colocalization between RHO^WT^ and ubiquitin was observed in merged images (Figure 5A1, G1). In contrast, in *Rho* ^*∆I256*^-EGFP transfected HEK293 cells, ubiquitin staining was found in numerous accumulated puncta (Figure 5F), which localized with RHO aggregates in the cytosol (Figure 5E1’, C1’). In COS-7 cells, the ubiquitin staining pattern was less punctated, more clustered (Figure 5L), and was mainly recruited to the RHO aggregates pool (Figure 5K1’, I1’). A similar ubiquitin pattern to that in *Rho* ^*∆I256*^-EGFP transfected was seen in *Rho* ^*P23H*^-EGFP transfected cells. Consistent with these immunostaining results, Pearson’s correlation coefficient analysis also revealed a significantly higher colocalization between RHO^∆I256^ (or RHO^P23H^) aggregates and Ub in contrast to RHO^WT^ (Supplementary Figure S3C).

Subsequently, we used immunoblotting to examine the expression pattern of ubiquitin in lysates of untransfected HEK293 cells and in cells transfected with different *Rho* constructs (untransfected cells were used as control) and demonstrated that similar to RHO^P23H^, RHO^∆I256^ is ubiquitinated to a greater extent than RHO^WT^. We detected a significantly increased intensity of the characteristic ubiquitin smear bands in lysates obtained from mutant *Rho* ^*∆I256*^ or *Rho* ^*P23H*^ transfected cells compared to those obtained from *Rho* ^*WT*^ transfected cells (Figure 5M), particularly evident in polyubiquitin chains larger than 180 kDa. The untransfected control cells also showed the same pattern of Ub expression, indicating that the ubiquitin signal is not due to overexpression. To quantify the expression of polyubiquitin protein, we compared the levels between WT transfected group and the mutant transfected groups (protein level higher than 20 kDa). The results showed that polyubiquitin was significantly increased in HEK293 cells expressing RHO^∆I256^ and RHO^P23H^ than in RHO^WT^ (Figure 5N). As ubiquitin acts as a tag that identifies proteins for ERAD-mediated degradation, our results indicate that ubiquitinated RHO^∆I256^ aggregates are likely to be a substrate of the ERAD pathway.

### 2.6 RHO^∆I256^ colocalizes with the proteasome 20S subunit

The process of ERAD culminates with the degradation of misfolded proteins by the ubiquitin-proteasome system (UPS). In this process, substrates are tagged with ubiquitin (Ub), form polyubiquitinated adducts, and are then recognized and degraded by the 26S proteasome (Bence et al., 2001; Adams, 2003; Schwartz and Ciechanover, 2009; Sen et al., 2021b). The 26S proteasome consists of one 20S protein subunit and two 19S regulatory cap subunits. The 20S core region is responsible for substrate degradation (Gomez-Tourino et al., 2013). To analyze proteasomal rhodopsin degradation, we used a specific antibody against the proteasome 20S subunit beta 5 (PSMB5) to visualize this subunit in HEK293 and COS-7 cells transfected with plasmids encoding *Rho* and to observe its colocalization with RHO^∆I256^ aggregates.

**FIGURE 6:**
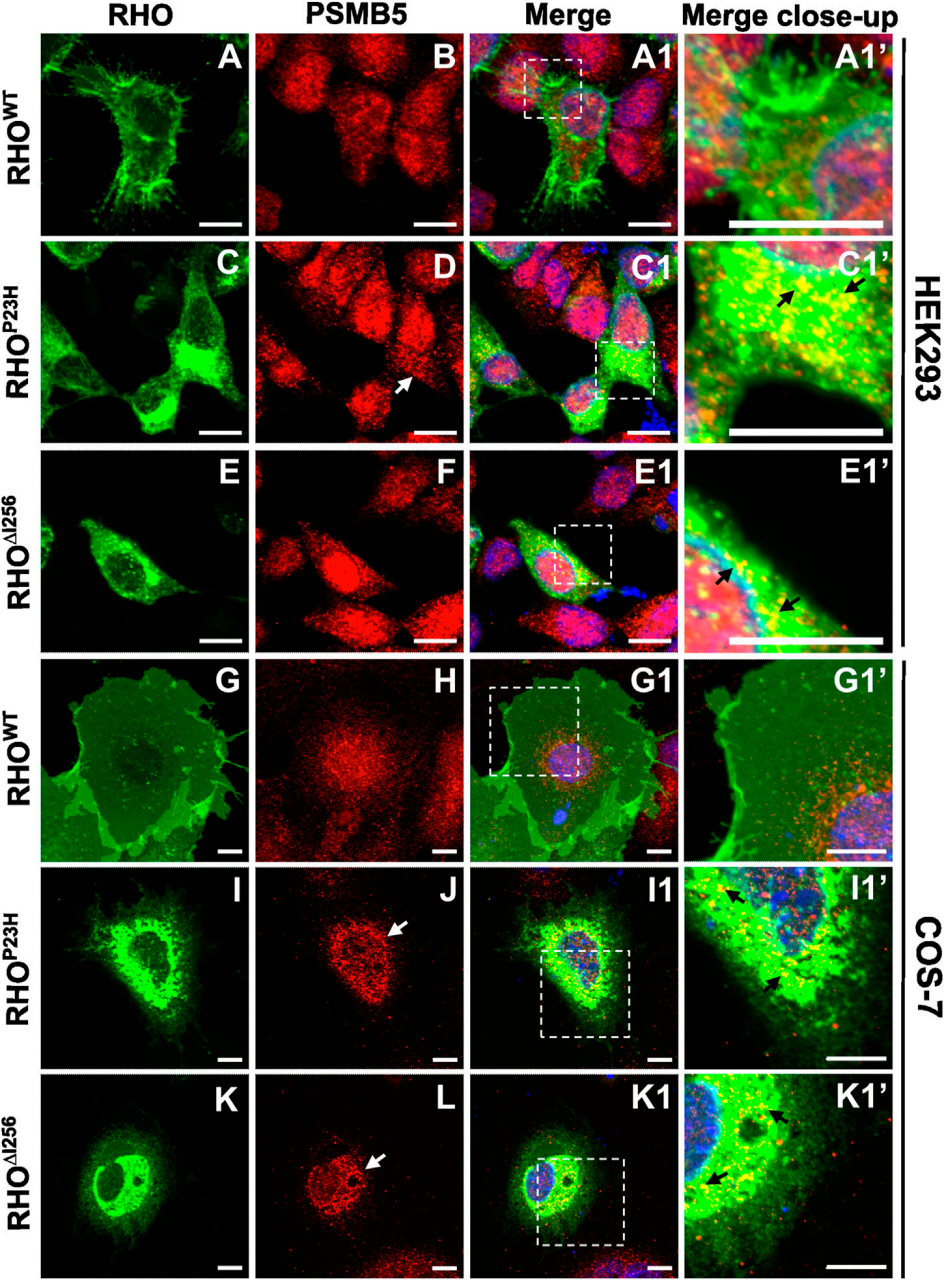
Rho^∆I256^ is targeted to the proteasome system. Co-staining of EGFP-tagged RHO^∆I256^, RHO^WT^, or RHO^P23H^ (green, first column) with PSMB5 (red, second column) in HEK293 **(A–E1’)** and COS-7 cells **(G–K1’)**. Colocalization is shown in merged pictures (third column). PSMB5 distributed in the cytosol and nucleus in *Rho* ^*WT*^ *-*EGFP transfected cells with no overlapping signal. In *Rho* ^∆I256^ *-*EGFP transfected cells, PSMB5 partially accumulated in the cytosol (white arrows in **F and L**) and colocalized with RHO^∆I256^ aggregates (black arrows in **E1’ and K1’**). Similar results were also seen in *Rho* ^*P23H*^ *-*EGFP transfected cells (white arrows in **D and J**; black arrows in **C1’, I1’**). Scale bar: 10 μm.

We found that RHO^∆I256^ aggregates were transported to the proteasome system and colocalized with PSMB5. In *Rho* ^*WT*^-EGFP-transfected HEK293 and COS-7 cells, PSMB5 staining was shown in the cytoplasm and the nucleus (Figure 6B, H). However, in RHO^∆I256^-EGFP-expressing cells, PSMB5 accumulated and formed perinuclear punctate aggregates in the cytosol. This phenomenon was more pronounced in COS-7 cells than in HEK293 cells (Figure 6D, F, J, L). Most importantly, the punctate distribution of PSMB5 overlapped with a fraction of perinuclear RHO^∆I256^ aggregates (Figure 6C1’, I1’, J1’, K1’). Pearson’s correlation coefficient analysis also showed a significantly greater colocalization between RHO^∆I256^ (or RHO^P23H^) aggregates and PSMB5 than RHO^WT^ (Supplementary Figure S3D). These results suggest that a fraction of these aggregates have already been retrotranslocated from the ER to the proteasome system, probably as a consequence of ERAD-mediated degradation.

### 2.7 Inhibition of VCP- and proteasomal activity increase the abundance of high molecular weight RHO aggregates *in vitro*

The colocalization of RHO^∆I256^ with different ERAD pathway markers suggested that the ERAD machinery may likely be involved in the clearance of RHO^∆I256^ aggregates. To verify this hypothesis further, we inhibited the proteasome or the chaperone VCP.

**FIGURE 7:**
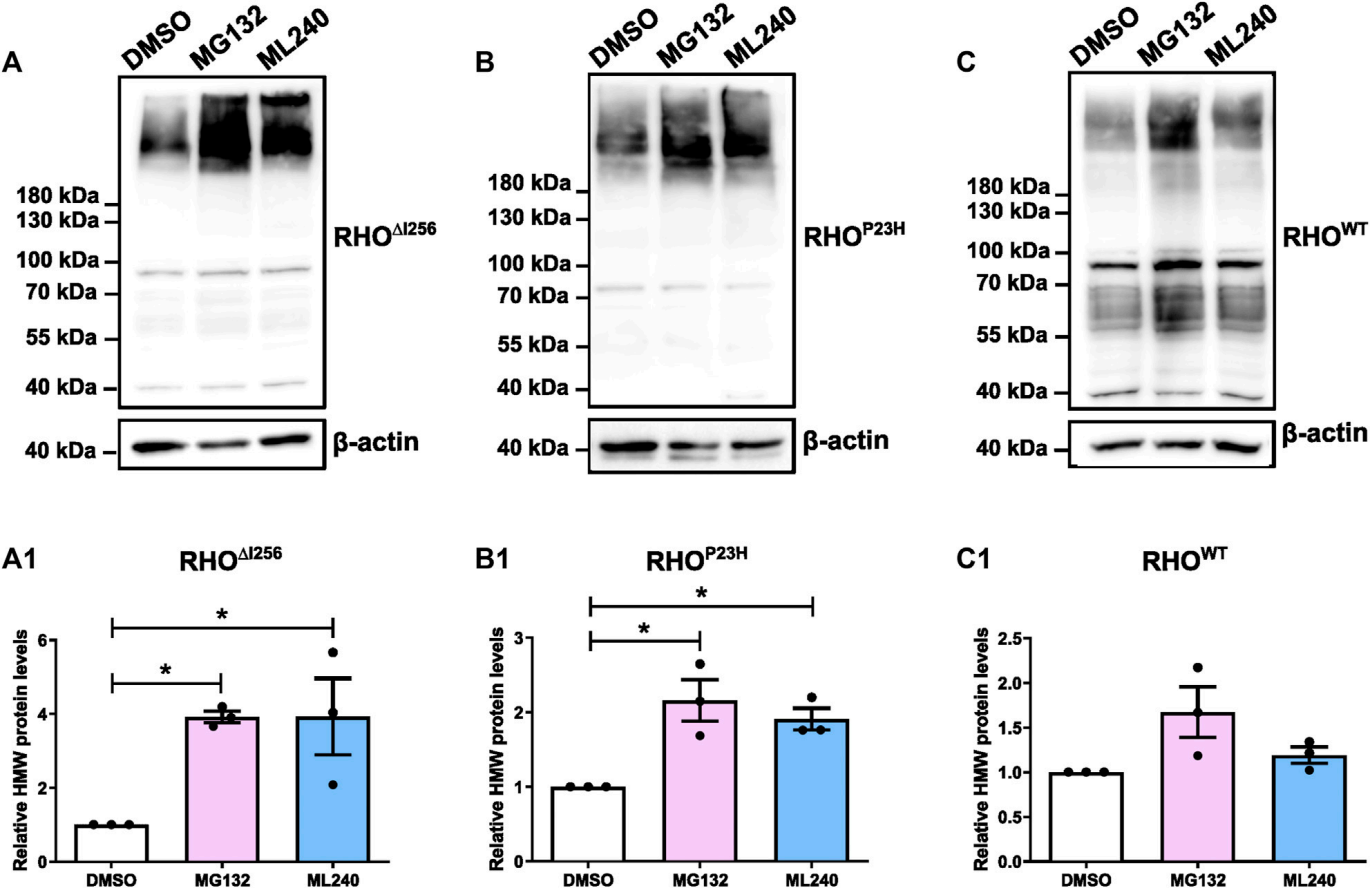
Proteasome and VCP inhibition increases HMW aggregates load in *Rho* ^*∆I256*^-transfected HEK293 cells. Western blot results for RHO in HEK293 cells after transfection with *Rho* ^*∆I256*^-EGFP **(A)**, *Rho* ^*P23H*^-EGFP **(B)**, or *Rho* ^*WT*^-EGFP **(C)** followed by treatment with DMSO, 5 µM MG132 or 2 µM ML240 for 6 h. The gray value changes in HMW rhodopsin aggregates bands above 180 kDa following ML132 or ML240 treatment relative to DMSO vehicle treatment is quantified and presented in **(A1, B1, and C1)**. β-actin serves as housekeeping protein control. Data were expressed as mean ± SEM. Significance was calculated by a one-way ANOVA and Bonferroni’s multiple comparisons test. *p < 0.05. Three independent experiments are included in this data analysis.

HEK293 cells overexpressing *Rho* ^*WT*^-EGFP, *Rho* ^*∆I256*^-EGFP, or *Rho* ^*P23H*^-EGFP were treated with the proteasome inhibitor MG132 (5 µM), the VCP inhibitor ML240 (2 µM) or vehicle (DMSO, 0.1%), for 6 h. Inhibition with MG132 or ML240 significantly increased the levels of HMW aggregates in the lysates of *Rho* ^*∆I256*^-EGFP transfected cells (Figure 7A). The average intensity of the bands corresponding to RHO HMW aggregates (molecular weight≥180 kDa), indicated that MG132 or ML240 significantly increased the amount of HMW proteins compared to the vehicle-treated group. These results suggest that both proteasomal and VCP activity are essential for the degradation of RHO^∆I256^ during the ERAD process (Figure 7A1).

The levels of RHO^P23H^ HMW aggregates also significantly increased after MG132 or ML240 treatment, which is consistent with previous findings (Figure 7B, B1) (Illing et al., 2002; Griciuc et al., 2010a; Griciuc et al., 2010b). In addition, the RHO^WT^ HMW aggregates increased slightly, suggesting that RHO^WT^, when overexpressed, also requires the proteasome for degradation (Figure 7C, C1).

## 3 Discussion

*Rho* mutations, a principal cause of adRP, result in changes in protein structure and function. They are classified into seven classes, each with varying defects, including misfolding and disruptions in proteostasis, mislocalization, disrupted intracellular trafficking, instability, and altered function (Athanasiou et al., 2018). The *Rho* ^*∆I256*^ mutation is a prevalent primary cause of adRP in a large British kindred as well as in patients from other Western European countries and China (Inglehearn et al., 1991; Artlich et al., 1992; Lorenz et al., 1993b; Li et al., 2010). So far, the precise mechanism and biochemical defects linking it to photoreceptor degeneration have remained unclear.

Our study reveals, for the first time, that a dominant-negative (DN) effect significantly contributes to *Rho* ^*∆I256*^-associated retinal degeneration. RHO^∆I256^ accumulates in the endoplasmic reticulum (ER) as a substrate for VCP-dependent ER-associated degradation (ERAD). Like RHO^P23H^, RHO^∆I256^ forms perinuclear aggregates, hindering RHO^WT^ from targeting the plasma membrane in cell models or reaching rod outer segments (ROS) in retinal explants. We show here that RHO^∆I256^ aggregates colocalize with VCP and undergo polyubiquitination before being retrotranslocated from the ER to the proteasome system. When inhibiting these processes pharmacologically, we observed decreased clearance of aggregates, indicating that both VCP and the proteasome play critical roles in the degradation of RHO^∆I256^ aggregates.

Biochemical analysis using HEK293 and COS-7 cells as models demonstrates that, unlike RHO^WT^ and very similar to RHO^P23H^, RHO^∆I256^ protein mislocalizes in the perinuclear region of the cell cytosol, failing to target the cell membrane. Detergent-soluble and detergent-insoluble lysate fractions revealed a higher tendency for RHO^∆I256^ than RHO^WT^ to form aggregates, including high molecular weight (HMW) aggregates exceeding 180 kDa, which is in line with previous studies (Sung et al., 1993). This again resembles aggregation patterns seen with RHO^P23H^ (Illing et al., 2002; Griciuc et al., 2010a). Yet, there is a higher tendency for RHO^∆I256^ to form HMW aggregates than RHO^P23H^. This finding goes in line with clinical observations that patients with *Rho* ^*∆I256*^ experience a more severe natural history of disease than those with *Rho* ^*P23H*^ (Inglehearn et al., 1991; Artlich et al., 1992; Lorenz et al., 1993a). This could be, at least in part, due to the fact that the deletion of one of the two isoleucine at position 255 or 256 within the sixth transmembrane domain of RHO shortens this intramembrane stretch significantly, affecting the subsequent 20 amino acids as well (Lorenz et al., 1993a).

Given that under normal conditions, RHO likely acts as a functional dimer, the impaired functionality of oligomeric aggregates is expected, hindering normal processes like G protein coupling, interaction with RHO kinase, and engagement with arrestin in the phototransduction cascade (Chabre and Le Maire, 2001; Whorton et al., 2007; Whorton et al., 2008). Nevertheless, the precise mechanisms of how these aggregates lead to cell death remain unknown. A dominant-negative (DN) effect of the mutant protein on the normal protein was reported to account for the dominance of other mutant *Rho* alleles in adRP inheritance (Illing et al., 2002; Saliba PMM et al., 2002; Wilson and Wensel, 2003; Griciuc, 2010). To explore whether RHO^∆I256^ has an effect on RHO^WT^, we conducted an experiment co-expressing EGFP-tagged *Rho* ^*∆I256*^ with mcherry-tagged *Rho* ^*WT*^ in cells at equivalent dosage, mirroring conditions observed in *RHO* ^*∆I256*^ heterozygous patients. The result revealed significant retention of RHO^WT^ protein in the cell cytosol, colocalizing with intracellular RHO^∆I256^ aggregates. Similar outcomes were observed in cells co-transfected with *Rho* ^*P23H*^ and *Rho* ^*WT*^, aligning with prior studies demonstrating that the *RHO* ^*P23H*^ mutation disrupts *RHO* ^*WT*^ processing, leading to protein inclusions within the cell and impeding RHO^WT^ delivery to the plasma membrane (Illing et al., 2002). In contrast, co-expression of *Rho* ^*WT*^-EGFP with *Rho* ^*WT*^-mcherry did not result in aggregates with both RHO^WT^ fusion proteins reaching the cell membrane. These findings confirm the dominant interference of both *Rho* ^*∆I256*^ and *Rho* ^*P23H*^ into overall RHO trafficking, suggesting the physical interaction between mutant and wild-type protein.

We extended our study to a three-dimensional retinal explant culture system to better evaluate the DN effect in a more biologically and clinically relevant model. We expressed exogenous *Rho* plasmids in C57BL/6J (WT) retinal explants using the Magnetofection™ method (Sen et al., 2021a; Bassetto et al., 2021). In WT retinae transfected with mutant *Rho* ^*∆I256*^-EGFP or *Rho* ^*P23H*^-EGFP plasmids, fluorescence emitted by *Rho* ^*∆I256*^-EGFP overlapped with that of RHO^WT^ protein in the ONL, contrasting with *Rho* ^*WT*^-EGFP-transfected retinae where both endogenous as well as exogenous RHO^WT^ accurately localized to the OS. These results demonstrate that mutant RHO influences the expression or trafficking of endogenous RHO^WT^, confirming the DN effect of *Rho* ^*∆I256*^ as well as that of *Rho* ^*P23H*^ in -adRP. Our findings on *Rho* ^*P23H*^ align with studies showing the interaction between RHO^P23H^ aggregates and RHO^WT^ through Förster resonance energy transfer (FRET) (Rajan and Kopito, 2005). Other publications affirm that while RHO^P23H^ does aggregate; however, there is no physical interaction with RHO^WT^ (Gragg and Park, 2018). Nonetheless, all studies, including ours, report colocalization between RHO^P23H^ and RHO^WT^ under microscopy, consistent with the notion that these different mutant RHOs hinder the correct trafficking of RHO^WT^ to the plasma membrane. ER retention of aberrant RHO aggregates is one typical feature of class II adRP (Athanasiou et al., 2018). *Rho* ^*∆I256*^ falls into this category, and we suggest it belongs to class II.

We found *Rho* ^*∆I256*^ to form ER-associated aggregates colocalizing with VCP, ubiquitin, and the proteasome system, three important components of the ERAD process. RHO^WT^ experiences repeated cycles of internalization-degradation (Satoh and Ready, 2005), and in our current study, RHO^WT^ accumulated after VCP and proteasome inhibition, suggesting that RHO^WT^ can also undergo ERAD degradation.

**FIGURE 8:**
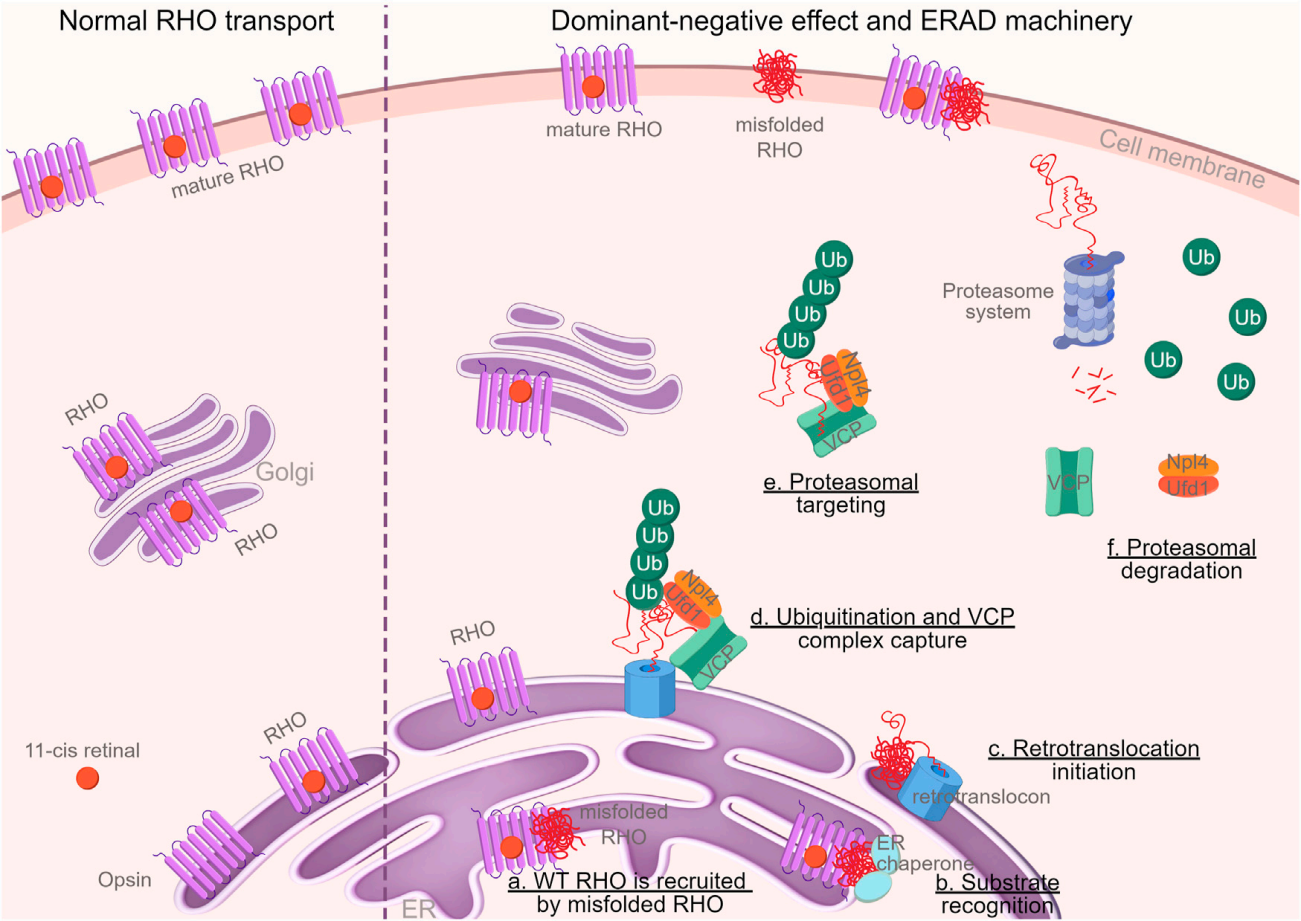
Graphical abstract. Schematic illustration of the dominant-negative mechanism of RHO^∆I256^ on RHO^WT^ and the degradation of these mutant proteins in the ERAD machinery (*By FigDraw, ID: TWOIA64640*). Misfolded RHO gets withheld in the ER and traps RHO^WT^ (a). Molecular chaperones, such as calnexin, calreticulin, and HSP70, identify misfolded proteins (b) and transport them to the ER retrotranslocation site, where the polypeptides are partially removed from the ER (c) and polyubiquitinated (d). Ubiquitination and VCP complex formation attract ubiquitin fusion degradation 1 (UFD1) and nuclear-protein localization 4 (NPL4) proteins to the retrotranslocon, which initiates the exit of the retrotranslocon from ER to cytoplasm (d). The complex retrotranslocates further into the cytoplasm (step d to e), where VCP-ATPase activity unfolds the protein (e). The unfolded VCP substrate is delivered to the proteasome for full degradation (f) (Vembar and Brodsky, 2008). Ubiquitin, VCP, and other cytosolic chaperones are then recycled.

In conclusion, our research provides novel evidence supporting a dominant-negative role of the mutant allele in *Rho* ^*∆I256*^-mediated retinitis pigmentosa. The observed formation and degradation of RHO^∆I256^ aggregates through the VCP-dependent ERAD pathway makes it a candidate for pharmacological therapy through VCP inhibition. The whole process was described in graphical abstract (Figure 8).

However, further validation in animal model systems is crucial to confirm both the classification of *Rho* ^*∆I256*^ as a class II adRP and a possible therapeutic option of targeting the VCP-dependent ERAD pathway for this form of adRP.

## 4 Materials and methods

### 4.1 Primers design, cloning, and *E. coli* transformation

Plasmids pEGFP-N1 encoding bovine *Rho (WT or P23H)* driven by the CMV promoter were provided by M. Cheetham (Saliba PMM et al., 2002). For the *Rho* ^*∆I256*^ mutation, we designed the primers (IDT primer quest tool), forward primer: 5’-CCG CAT GGT CAT CAT GGT CAT CG-3’ and reverse primer: 5’-CAG GAA AGC GAT GAC CAT GAT GAC CAT GC-3’. *Rho* ^*∆I256*^ was obtained and amplified by PCR using bovine *Rho* ^*WT*^ as template. To harvest single colonies that contained *Rho* ^*∆I256*^, chemical transformation of competent *E. coli* was performed with 2.5 µL of PCR reaction product added into 50 µL of cells. After that, plasmid DNA was prepared from small-scale (1.5 mL, miniprep) bacteria cultures using QIAprep Spin Plasmid Miniprep Kit or from large-scale (50 mL) overnight cultures using the PureYield Plasmid Midiprep System. (*Notes: Isoleucine residues at codons 255 and 256 are conserved in bovine and human Rho. So, we designed the deletion of isoleucine at codon 256 based on the wild-type bovine Rho backbone in the pEGFP vector and applied it in our study.*)

### 4.2 Cell culture and transfections

HEK293 human embryonic kidney cells (CRL-1573, ATCC—Global Bioresource Center) and COS-7 African green monkey kidney fibroblast-like cells (CRL-1651, ATCC—Global Bioresource Center) were cultured in DMEM supplemented with 10% heat-inactivated fetal bovine serum and 1% penicillin/streptomycin (Thermofisher Scientific). In addition, cells were transiently transfected with 1 µg of *Rho* expression plasmids using TransIT-LT1 transfection reagent (MIR2300, Mirus). HEK293 and COS-7 cells were cultured on the Poly-D-lysine (P6407, Sigma) coating 24 well plates with autoclaved glass coverslips (1.2 cm diameter) for immunostaining or 6 well plates without coverslips for Western blot. Cells were transiently transfected with *Rho-EGFP* constructs or co-transfected with *Rho-EGFP* and *Rho* ^*WT*^ *-mcherry* after 80% of cell confluency and incubated for 48 h.

### 4.3 Animals

C57BL/6J mice were obtained from Jackson Laboratory (JAX stock #000664, RRID: IMSR_JAX:000664) and housed in the animal facility of the Tübingen Institute for Ophthalmic Research under standard white cyclic lighting, had free access to food and water, and were used irrespective of gender. All procedures were performed in accordance with the Association for Research in Vision and Ophthalmology (ARVO) declaration for the use of animals in ophthalmic and vision research and the law on animal protection issued by the German Federal Government (Tierschutzgesetz) and were approved by the institutional animal welfare office of the University of Tübingen. All efforts were made to minimize the number of animals used and their suffering.

### 4.4 Immunofluorescence and microscopy

Cells were fixed and stained 48 h after transfection, as described before (Mendes and Cheetham, 2008). Samples were incubated overnight at 4°C with the primary antibodies (calnexin: C5C9, Rabbit monoclonal, 1:100, Cell signaling; VCP: mouse monoclonal, 1:200, Invitrogen; ubiquitin: sc8017, mouse monoclonal, 1:50, Santa Cruz; or proteasome subunit 20S: PSMB5, rabbit polyclonal, 1:100, Invitrogen followed by incubation with secondary antibodies (IgG Alexa Fluor™ 568 or 660 dye-conjugated goat anti-mouse or anti-rabbit respectively, Molecular Probes, Inc. Eugene, United States of America). Negative controls were carried out by omitting the primary antibody. To measure RHO immunofluorescence intensity, the whole cell body of HEK293 cells (n ≥ 40) and COS-7 cells (n ≥ 20) of three seperate experimental replicates were carefully contoured, and the fluorescence intensity value was automatically measured by ZEN software. Colocalization analysis using Pearson’s correlation coefficient between RHO and ERAD markers was performed using ZEN software. We select at least 6 distinct regions on the cell coverslip for data analysis. All images were captured and analyzed using a Zeiss Axio Imager Z1 ApoTome microscope, AxioCam MRm camera, and Zeiss Zen 2.3 software at 20X or ×63 magnification.

### 4.5 VCP- and proteasome inhibition assay

HEK293 cells were incubated with 5 µM proteasome inhibitor MG132 (J63250, Alfa Ascar, Thermo Fischer Scientific) or 2 µM VCP inhibitor, ML240 (5,153, Tocris) dissolved in 0.1% DMSO for 6 h after 48 h of transfection. For each inhibitor, 0.1% DMSO was applied as vehicle control. Cell lysates were then harvested and analyzed by Western blot.

### 4.6 Western blot

HEK293 cells transfected with *Rho-EGFP* were harvested after 48 h of incubation and then lysed at 4°C in 0.5% Nonident P40 (NP40, Roche) lysis buffer with 1% phosphatase inhibitor cocktail 2 and 3 (PI2 and PI3, Sigma), 2% protease inhibitor complex (PIC, Roche) in TBS. Lysates were centrifuged at 16,000 *g* for 15 min at 4°C. For detergent-insoluble fractions, pellets were solubilized in 1% SDS in PBS at RT, and lysis buffer was added. The pellets were then sonicated at 4°C, and the fraction mixtures were incubated 30 min and then centrifuged at 100* g* for 10 min, both at 4°C (Griciuc et al., 2010b). For detergent-soluble/-insoluble samples, protein concentration was determined by Bradford assay kits. Cell lysates were normalized for total protein, and 7.5 µg (RHO protein in detergent-soluble), 10 µg (RHO in detergent-insoluble fractions), or 20 µg (Ub protein or RHO protein after VCP inhibition) total protein was prepared and mixed with 1x Laemmli sample buffer before being separated by 8%–16% Tris-Glycine precast gels (Invitrogen) and electroblotted onto PVDF membranes. Immunoblotting was performed according to standard protocols using the as primary antibodies: anti-RHO (1D4, MAB5316, 1:300, Sigma), anti-VCP (MA3-004 1:1,000, Invitrogen), anti-ubiquitin (sc8017, 1:500, Santa Cruz), and anti-β-actin (3700S 1:2000, cell signaling). Afterward, horseradish peroxidase-coupled (HRP) secondary antibodies were applied (1:2000, cell signaling). The ECL Plus chemiluminescent and chemifluorescent HRP detection reagent (Thermo Scientific™ Pierce™ ECL) was used according to the manufacturer’s instructions. Band intensity was quantified by *ImageJ* software, and relative protein expression levels were determined based on the WT or untreated control group.

### 4.7 Organotypic culture preparation and classic magnetofection

*In vitro* retina cultures were prepared according to published protocols (Arango-Gonzalez et al., 2010). PN9 animals were sacrificed, and the eyeballs were removed and collected in a serum-free R16 culture medium (07490743A, Invitrogen Life Technologies). The eyes were pretreated with 0.12% Proteinase K (0219350490, MP Biomedicals) for 15 min at 37°C in R16 medium. After enzymatic digestion of the eyeball, the retina and the attached pigment epithelium were dissected, and four radial cuts were made to flatten it. Retinas were placed on culture plate inserts (CLS3412, Corning Life Sciences) with the ganglion cell layer uppermost. The retinal explants were cultured in 1 mL serum-free culture medium consisting of Neurobasal A (21103049, Thermo Fischer Scientific) supplemented with 2% B-27 supplement (17504044, Thermo Fischer Scientific), 1% N2 supplement (17502048, Thermo Fischer Scientific), 1% penicillin solution (15140-122, Thermo Fischer Scientific), and 0.4% GlutaMax (35050061, Thermo Fischer Scientific) and maintained at 37°C in a humidified atmosphere with 5% CO_2_. At the same time, 2 µg EGFP-tagged *Rho* ^*WT*^, *Rho* ^*P23H*^, *Rho* ^*∆I256*^ plasmids, and 1.5 µL magnetic nanoparticles (XPMag Explant Transfection Reagent, OZBiosciences) were complexed for 30 min in Neurobasal-A medium, to a total volume of 10 µL. The solution was incubated at room temperature for 30 min to allow the cationic magnetic nanoparticles to bind to the negatively charged DNA plasmids. After complexation, the magnetic complexes were administered to the top of each retina (on the ganglion layer side). The super-magnetic plate was immediately placed under the 6-well plate. Then, the set well plate/magnet was kept in the incubator for 1 h, to allow the pull down of the complexes through the retinae. After removal of the magnet, retinae in the 6-well plates remained incubated at 37°C in 5% CO_2_ for 3 days before fixation in 4% paraformaldehyde.

### 4.8 Preparation and immunohistochemistry on retinal sections

The explants were immersed in 4% paraformaldehyde in 0.1 M phosphate buffer (PB; pH 7.4) for 45 min at RT, followed by cryoprotection in graded sucrose solutions (10%, 20%, 30%) and embedded in cryomatrix (Tissue-Tek^®^ OCT Compound, VWR). Radial sections (14 µm) were collected, air-dried, and stored at −20°C. Retina sections were incubated overnight at 4°C with rhodopsin primary antibody (1D4, MAB5316, 1:300, Sigma) followed by incubation with secondary antibody (Alexa Fluor™ 568 dye-conjugated goat anti-mouse IgG). DAPI (D9542, Sigma) was used as a nuclear counterstain. Finally, the slides were mounted with Fluoromount-G Mounting Medium (17984-25, EMS).

### 4.9 Statistical analyses

Mann-Whitney-U-test analysis was used to examine RHO MFI that did not match Gaussian distribution; while, Pearson’s correlation coefficient and protein gray levels that were consistent with normal distribution were analyzed using one-way ANOVA followed by Bonferroni’s *post hoc* comparisons tests in *GraphPad Prism 9* software. Data were presented as mean ± standard error of the mean (SEM). Differences were considered significant and indicated as *p < 0.05, **p < 0.01, ***p < 0.001, ****p < 0.0001.

## Data Availability

The original contributions presented in the study are included in the article/Supplementary Material, further inquiries can be directed to the corresponding authors.
