# Supplementary material for: Mutant dominant-negative rhodopsin ∆I256 causes protein aggregates degraded via ERAD and prevents normal rhodopsin from proper membrane trafficking
Source: Front Mol Biosci. 2024 May 17;11:1369000. doi: 10.3389/fmolb.2024.1369000 (PMC11140085; doi:10.3389/fmolb.2024.1369000)
Supplement: Supplementary file 1 [file DataSheet1.PDF]

Supplementary Material

Supplementary Tables

Table S1. List of abbreviations

Abbreviation	***Definition***
Rho	rhodopsin gene
RHO	rhodopsin protein
WT	Wild-type
P23H	proline 23 to histidine
ΔI256	Isoleucine 255/256 deletion
adRP	autosomal dominant retinitis pigmentosa
DN	dominant-negative
VCP	valosin containing protein
ERAD	endoplasmic reticulum-associated degradation
Ub	Ubiquitin
UPS	ubiquitin-proteasome-dependent system
UPR	unfolded protein response
HMW	high molecular weight
XPMag	magnetic nanoparticles

Table S2. List of six different magnetofection groups

Groups \ Treats	*Rho*^{WT}-EGFP Plasmid	*Rho*^{P23H}-EGFP Plasmid	*Rho*$^{\Delta I256}$-EGFP Plasmid	XPMag	pEGFP empty vector
CM	-	-	-	-	-
XPMag	-	-	-	+	-
EGFP	-	-	-	+	+
WT-EGFP	+	-	-	+	-
P23H-EGFP	-	+	-	+	-

ΔI256-EGFP	-	-	+	+	-

CM group: retinae received only medium and magnetic field exposure; **XPMag group**: retinae that received classic magnetofection but with 1.5 µl XPMag alone; **EGFP group**: retinae that treated with the 2 µg pEGFP empty vector combined with XPMag; **WT-, P23H- or ΔI256-EGFP** group: retinae that treated with classic magnetofection using 2 µg *Rho*^{WT}-EGFP, *Rho*^{P23H}-EGFP or *Rho*$^{\Delta I256}$-EGFP plasmids complexed with XPMag.

Supplementary Figures

Fig. S1

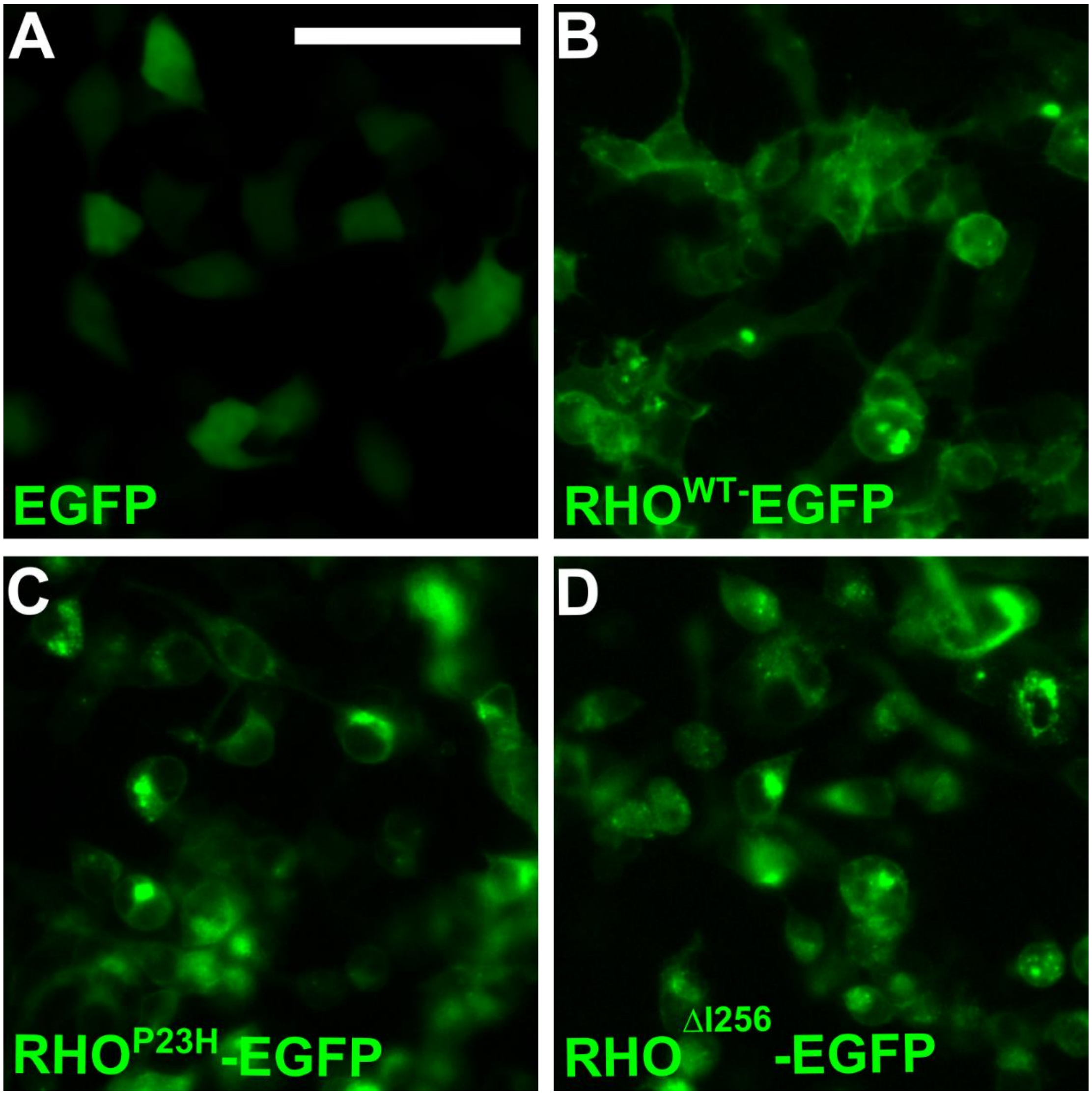

Fig. S1 Fluorescence pictures reveal the expression behavior of RHO in HEK293 cells that transfected with pEGFP plasmids encoding only EGFP (A), ***Rho***$^{\mathbf{WT}}$ **(B),** ***Rho***$^{\mathbf{P23H}}$ **(C) or** ***Rho***$^{\mathbf{\Delta I256}}$ **(D).** Cells transfected with the plasmid encoding only EGFP did not exhibit aggregate formation, while

RHO^{P23H} or RHO$^{\Delta I256}$ formed substantial cytosol aggregates. The majority of RHO^{WT} was correctly targeted at the plasma membrane. Scale bar: 20 µm.

Fig. S2

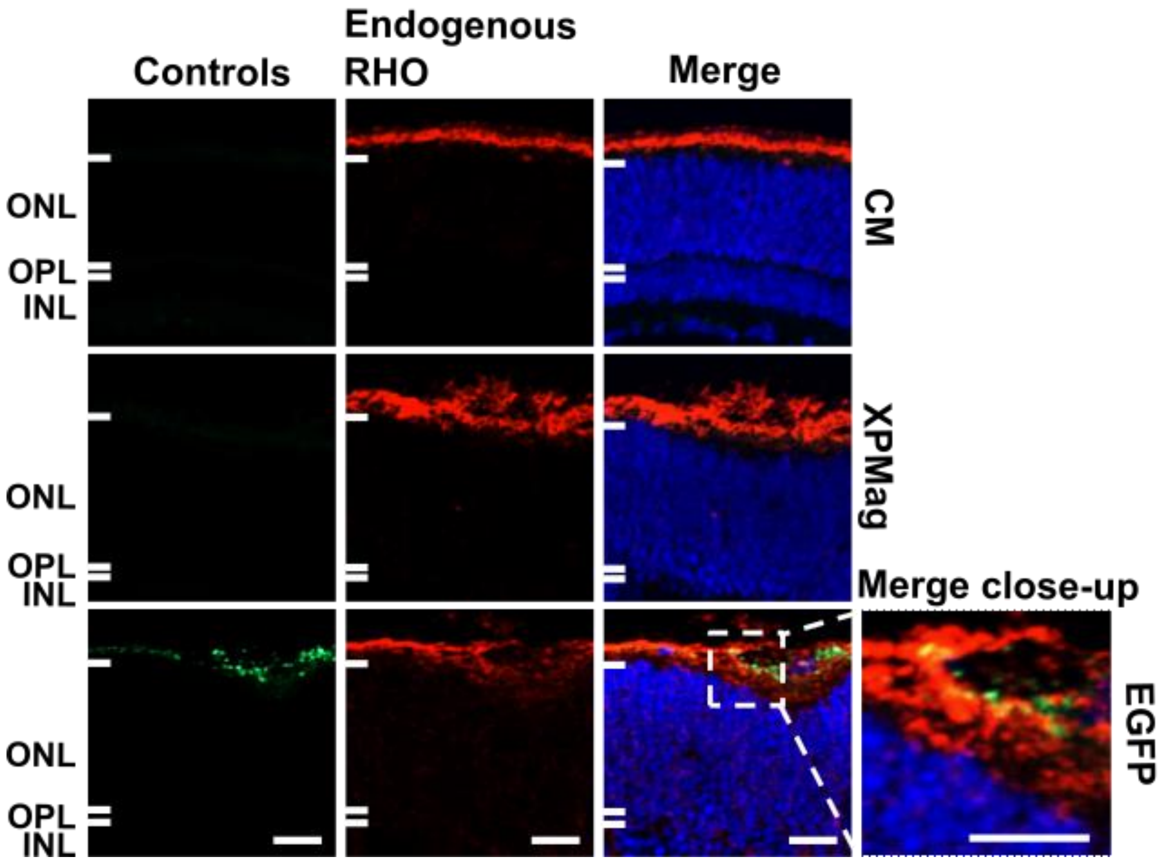

Fig. S2 Negative control of XPMag and EGFP controls in C57BL/6J retinal explants. Immunofluorescence pictures reveal the localization of endogenously expressed RHO in three control sample groups in C57BL/6J retinae (red, second column): CM treated (first row), XPMag transfected (second row), and EGFP/XPMag transfected (third row). Exogenous transfection of XPMag or empty pEGFP plasmid does not affect the expression and localization of endogenous wild-type RHO. In EGFP/XPMag transfected explants, the EGFP protein expression in the subretinal space does not colocalize with endogenously expressed RHO (merge close-up picture). Scale bar: 20 µm.

Fig. S3

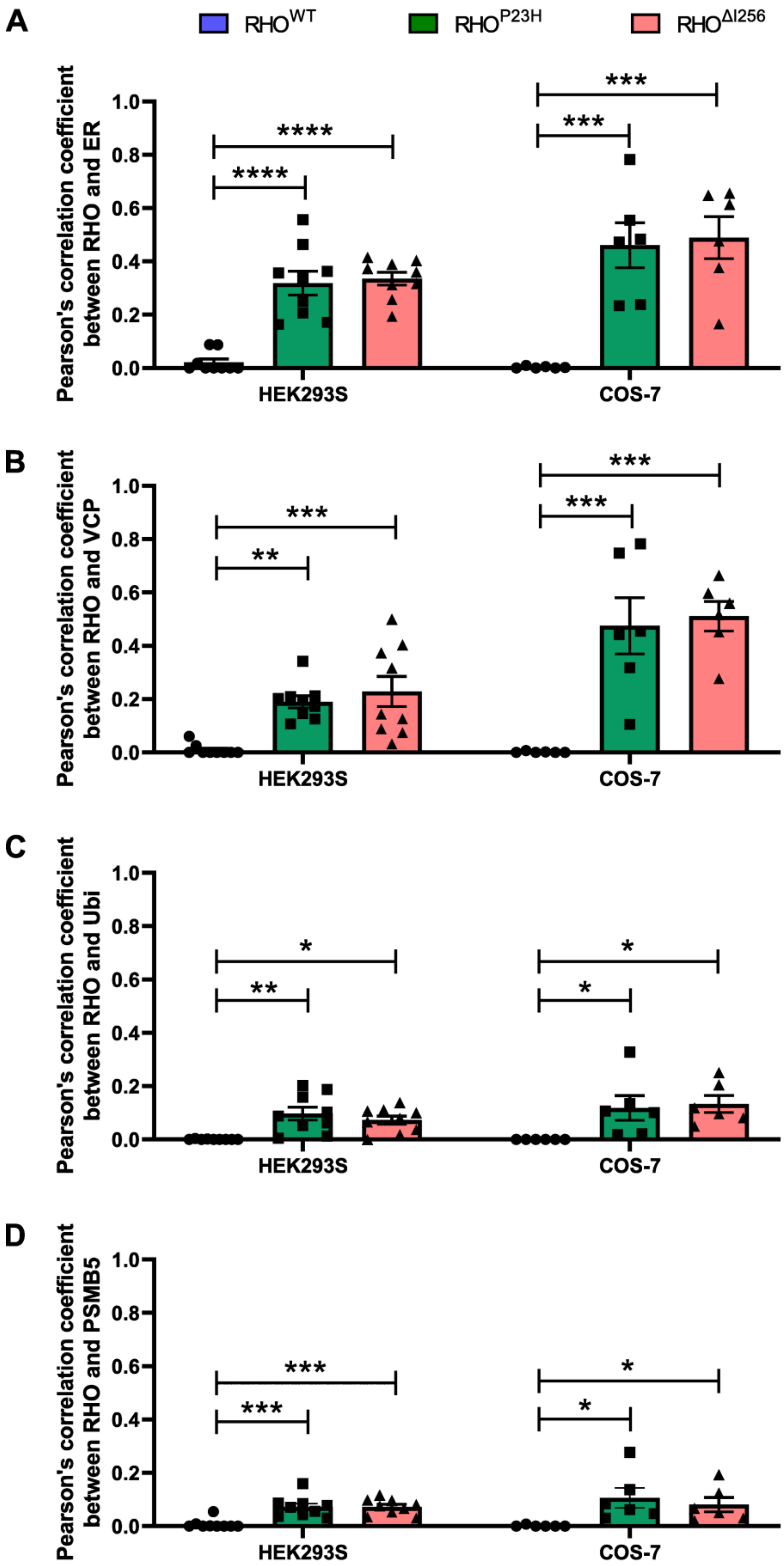
A
RHO^WT
RHO^P23H
RHO^ΔI256
Pearson's correlation coefficient between RHO and ER
HEK293S
COS-7
B
Pearson's correlation coefficient between RHO and VCP
C
Pearson's correlation coefficient between RHO and Ubi
D
Pearson's correlation coefficient between RHO and PSMB5

Fig. S3 Pearson's correlation coefficients between RHO and ERAD markers. The Pearson's correlation coefficients are used to quantify the co-localization between RHO and ER (A), VCP (B), ubiquitin (C), and PSMB5 (D). RHO^{P23H} and $RHO^{\Delta I256}$ aggregates have substantially greater correlation with ERAD markers than does RHO^{WT} protein. We select at least 6 distinct regions on cell coverslip for analysis. Data are expressed as mean ± SEM and significance was determined by one-way ANOVA followed by Bonferroni's multiple comparison test (*p<0.05, **p<0.01, ***p<0.001, ****p<0.0001).
